# Lethal Viral Sepsis/Septic Shock in Getah Virus-Infected *Ifnar1*−/− Mice

**DOI:** 10.3390/pathogens15101079

**Published:** 2026-10-14

**Authors:** Cameron R. Bishop, Bing Tang, Daniel J. Rawle, Thibaut Larcher, Andreas Suhrbier

**Affiliations:** 1 Infection and Inflammation Department, QIMR Berghofer, Brisbane, QLD 4029, Australia; bing.tang@qimrberghofer.edu.au (B.T.); daniel.rawle@qimrberghofer.edu.au (D.J.R.); 2 School of Chemistry and Molecular Biosciences, Faculty of Science, University of Queensland, St Lucia, QLD 2072, Australia; 3 APEX, UMR 703 PAnTher (INRAE/Oniris), 44307 Nantes, France; thibaut.larcher@inrae.fr; 4 GVN Center of Excellence, Australian Infectious Disease Research Centre, Brisbane, QLD 4029 and 4072, Australia

**Keywords:** Getah virus, *Ifnar1*−/− mice, RNA-Seq, viral sepsis, septic shock, cytokine storm, type I interferon, piglet

## Abstract

Getah virus (GETV) is a mosquito-borne Old World alphavirus that has recently increased its geographic range, particularly in China. GETV causes rheumatic disease in horses, but in recent years its primary impact has been lethal infections in newborn piglets. Herein, we characterize a lethal *Ifnar1*−/− mouse model of GETV which recapitulates many of the features seen in piglets. These include lethality, high viremia, diarrhoea, and splenomegaly, with histopathological findings aligning with those seen in piglets and indicating severe, acute, multi-organ inflammatory immunopathology. RNA-Seq of spleens from GETV-infected *Ifnar1*−/− mice and subsequent bioinformatic analyses illustrated a cytokine storm response that included robust type I interferon signatures and a pattern of cytokine/chemokine signatures indicative of viral sepsis/septic shock. *Ifnar1*−/−mice thus offer a framework and a mouse model for understanding GETV disease in piglets. This model also suggests that viral sepsis/septic shock may be the cause of lethality in GETV-infected piglets.

## 1. Introduction

Getah virus (GETV) is a mosquito-transmitted alphavirus found predominantly in South and East Asia [1,2]. GETV belongs to the Semliki Forest virus antigenic complex [3], which includes the Australasian Ross River virus (RRV) [4] and the globally distributed chikungunya virus (CHIKV) [5]. The geographic range of GETV is growing, in particular in China [2,6], with GETV contamination of a live porcine reproductive and respiratory syndrome virus vaccine potentially contributing [7]. Serosurveys suggest that a range of wild and domestic ungulates (hooved animals in the clade *Euungulata*) are the primary targets of infection by GETV [8]. The virus has caused disease outbreaks, leading to significant economic losses, primarily in horses and pigs [1,9,10,11,12]. Although human infection with GETV is frequently claimed, the contention arises from a small number of primary references that are somewhat dated [13,14,15,16], with no recent studies emerging that use modern, and substantially more reliable, serodiagnostic methodologies [17]. To the best of our knowledge, a confirmed clinical GETV infection in humans has not been reported. Cross-reactivity with other serologically related alphaviruses can also provide false seropositive results for GETV. For instance, historical serosurveys did suggest that GETV circulates in Australia; however, a series of analyses indicated that this is likely not the case, with serosurveys probably compromised by strong cross-reactivity of RRV immune sera with GETV [18].

GETV belongs to a group of Old World alphaviruses, such as RRV and CHIKV, where human symptomatic infections are usually associated with a spectrum of rheumatic disease manifestations, primarily fever, rash, myalgia and polyarthritis/polyarthralgia [4]. Symptomatic GETV infections in horses are also associated with fever, rash, hind limb oedema (mainly fetlock), and lymph node swelling lasting for 1–2 weeks [19]. GETV infection, like RRV [20], can affect racehorse performance, with a whole-virus formalin-inactivated combined vaccine for Japanese encephalitis virus (JEV) and GETV (JE/GETV vaccine) commercially available in Japan (Nisseiken Co., Ltd., Tokyo, Japan) for use in racehorses [3,21,22,23].

In contrast to GETV infection in horses, infection in neonatal and suckling piglets is often associated with mortality, preceded by a series of symptoms including fever, loss of appetite, diarrhoea, ataxia and cyanosis [11,24,25]. Increased virulence with 100% mortality [26,27] has recently been reported and was associated primarily with genotype III viruses [6,25,28,29]. To date, four GETV genotypes have been described based on the E2 sequence [1], with all genotypes likely belonging to a single serotype [30]. Fatal GETV infections are characterized by a constellation of histopathological lesions across multiple organ systems. In the lungs, GETV infection caused widened alveolar septa, epithelial desquamation, alveolar wall thickening, and intra-alveolar haemorrhage with leukocyte infiltration [11,24,27,31]. In the spleen, haemorrhagic splenitis was observed, characterized by reduced white-pulp volume, haemorrhage, and inflammatory cell infiltration [11]. In the liver, leukocyte infiltration, hepatocellular degeneration and haemorrhage were reported [11]. In the brain, mild neuronal degeneration and vascular inflammatory cell infiltration were seen [11]. In the kidneys, histopathology included inflammatory infiltrates with erythrocyte extravasation, glomerular vasodilation, tubular epithelial swelling, and irregular tubular lumina [11]. For intestines, villous epithelial injury with inflammatory lesions was reported by several groups [11,27,31]. These findings indicate an acute, severe multi-organ inflammatory immunopathology.

The GETV viremia levels in adult pigs are relatively low, peaking at ≈4 log_10_CCID_50_/mL [32], similar to adult wild-type mice [3]. In newborn piglets, the blood virus load would appear to be much higher, up to ≈7 log_10_ viral copies per ml [31], and (as noted above) is often associated with mortality. A key reason why neonatal piglets readily succumb to GETV infections is likely their diminished ability to produce type I interferon (IFN) in response to certain virus infections [33,34]. Mortality is also well described for alphavirus infections, including GETV infections in wild-type neonatal mice, again likely due to a diminished ability to generate type I IFN responses [35,36], as well as a permeable blood–brain barrier that can lead to brain infections and paralysis [37,38]. The early and rapid innate type I IFN response represents a key mechanism that protects more mature animals from excessive viremias and mortality [39,40,41,42,43,44,45]. The mechanisms underpinning poor type I IFN responses in piglets have not been well characterized. However, published mechanisms for murine and human neonates include impaired IFNα/β production in plasmacytoid dendritic cells [35], associated with reduced IRF7 activation [36,46,47], and impaired IFNβ production in monocyte-derived dendritic cells, associated with reduced IRF3 activity [48].

A range of efforts are underway to develop new GETV vaccines [22,49,50], antivirals [51,52], and diagnostics [53,54]. We thus sought to characterize a mouse model of GETV infection that recapitulates the lethal infection seen in young piglets. The use of neonatal piglets and neonatal mice for evaluating new interventions is relatively expensive, ethically challenging and often inconvenient, as pregnant animals give birth to variable numbers of offspring, often with timing that is difficult to synchronise. We therefore characterized adult, type I IFN receptor 1 knock-out (*Ifnar1*−/−) mice as a potential model of lethal GETV infection, as these animals emulate the defective type I IFN responses often observed in neonates, albeit via a different underpinning mechanism (see above). They also represent a widely available and widely used, well characterized, genetically modified mouse strain [43]. Cells in *Ifnar1*−/−mice are unable to respond to cytokine signalling mediated by IFNβ and a series of IFNαs [55], with these latter type I IFNs strongly induced by alphavirus infections in wild-type mice [44,56]. Infection of *Ifnar1*−/− mice (or other type I IFN response-deficient mice) with most pathogenic alphaviruses, including GETV [57,58], generally results in high viremias, a surge in pro-inflammatory cytokines, and mortality within a few days [40,41,42,43,59]. To provide a detailed characterization of the cytokine responses, we describe an RNA-Seq analysis of the spleens of GETV-infected *Ifnar1*−/− mice. Although RNA-Seq data have been reported for orthoflavivirus-infected *Ifnar1*−/− mice [60,61], to the best of our knowledge, RNA-Seq data from alphavirus infected *Ifnar1*−/− mice have not previously been reported and reveal surprisingly robust type I IFN responses despite the absence of a functional type I IFN receptor.

## 2. Materials and Methods

### 2.1. Ethics and Regulatory Statement

All mouse work was conducted in accordance with the “Australian code for the care and use of animals for scientific purposes” as defined by the National Health and Medical Research Council of Australia. Mouse work was approved by the QIMR Berghofer Animal Ethics Committee (P3746, A2108-612), first approved on 8 January 2025, with subsequent amendments approved through 22 January 2026. Breeding and the use of GMO mice were approved by the QIMRB Safety Committee under a Notifiable Low Risk Dealing (NLRD) Identifier: GTSC_075_2025: NLRD1.1(a). Research with live GETV was approved under the Queensland Biosecurity Act 2014, Scientific Research (prohibited matter)—Permit no. PRID000907. This legislation declares GETV to be an exotic virus [18], with specific containment and reporting procedures required to work with this virus in Queensland. All research was approved by the QIMR Berghofer Institutional Safety Committee.

### 2.2. The GETV Isolate, Culture and Titration

GETV_MM2021_ (GenBank MN849355) was obtained from the Doherty Virus collection, located at QIMR Berghofer. GETV_MM2021_ is the original 1955 prototype genotype I GETV isolate from Malaysia imported into Australia in the 1960s [18]. The virus was propagated in C6/36 cells and the titre determined using CCID_50_ assays, as described in [22].

### 2.3. Mouse Infection and Monitoring

*Ifnar1*−/− mice on a C57BL/6J background [62] were originally provided by Dr P. Hertzog (Monash University, Melbourne, Australia) and were bred in-house at QIMRB [57,63]. These mice do not express a functional Ifnar1 protein and are thus unable to signal via the IFNα/β receptor. Details of mouse agistment conditions have been described previously [22], with sibling male mice housed together and male mice from different litters housed separately. Mice were injected subcutaneously on the top/side of each hind foot [42] with 40 µL per foot (see [3] for details), with either tissue culture supernatant containing infectious GETV or with heat-inactivated (56 °C for 1 h) tissue culture supernatant containing inactivated GETV (Control). Mice were weighed and monitored daily [64] and were euthanized using CO_2_ when ethically defined endpoints were reached. Weight loss reaching or approaching 20% was the main criterion triggering the ethical requirement for euthanasia. Criteria were stipulated by the QIMR Berghofer Animal Ethics Committee for the approved project, P3746. In total, 5 male mice and 4 female mice were infected, and 5 male and 4 females were used as controls.

### 2.4. Histology

Histology was undertaken as described in [22,65] using a cohort of female mice. In brief, tissues were fixed in 10% formalin and embedded in paraffin (Sigma-Aldrich, St. Louis, MO, USA). Sections were stained with hematoxylin and eosin (H&E) (Sigma-Aldrich). Slides were scanned using Aperio AT Turbo (Aperio, Vista, CA, USA) and were viewed using Aperio ImageScope (v12.4.0.5043). Scanned slides were examined by a European board-certified veterinary pathologist using QuPath software v0.5.1 [66].

### 2.5. RNA-Seq and Bioinformatics

RNA-Seq was undertaken as described in [67,68,69] using a cohort of male mice. In brief, spleens (unperfused) were rapidly excised, cut open and placed in RNALater (Invitrogen, Carlsbad, CA, USA), with RNA subsequently extracted using TRIzol (Invitrogen). The RNA Integrity Number for one sample was too low for further processing, leaving GETV-infected mice *n* = 5 and Controls *n* = 4. Extracted RNA cDNA libraries were generated using the Illumina TruSeq Stranded mRNA library prep kit (polyA enrichment) (Illumina, San Diego, CA, USA). Sequencing was undertaken using the Illumina Nextseq 2000 platform to generate 75 bp paired end reads. STAR aligner was used to align the RNA-Seq reads to a combined mouse (GRCm39, version M27) and GETV_MM2021_ (GenBank MN849355) reference genome. A standard filter of 0.25 counts per million was applied. Differentially expressed genes (DEGs) were identified using EdgeR with a false discovery rate (q) cut-off of q < 0.01 and fold change > 2 (i.e., log_2_ > 1). These latter filters were applied, as described previously for CHIKV infections [56,62], to reduce DEG numbers that approach the range “for best results” from Ingenuity Pathway Analysis (IPA) [70]. The latter was performed using IPA, v84978992 (QIAGEN GmbH, Hilden, Germany) [69].

### 2.6. Statistics

Mouse data: The t-test (with Welch’s correction) was used if the difference in variances was <4 fold; t-test significance and variance were determined using Microsoft Excel. Otherwise, the non-parametric Kolmogorov–Smirnov exact test was performed using GraphPad Prism 10.

## 3. Results

### 3.1. GETV Virus Infection of Ifnar1−/− Mice, Weight Loss, Viremia and Haematocrit

**Figure 1:**
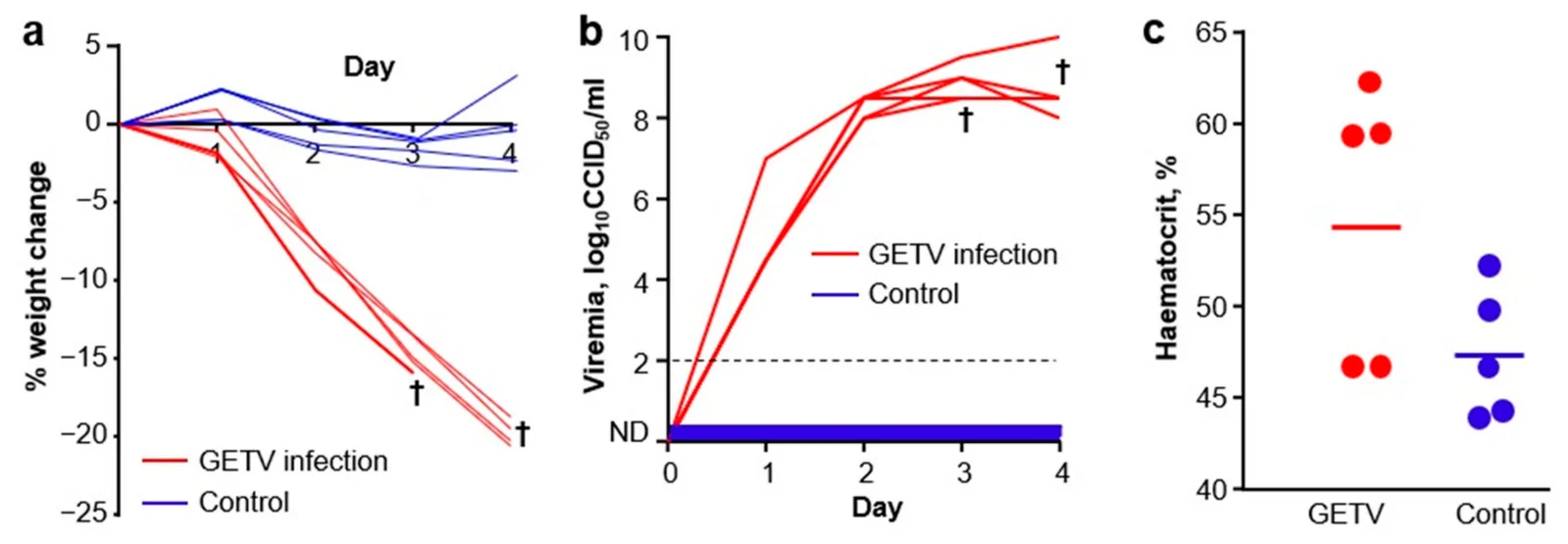
GETV infection of *Ifnar1*−/− mice. *Ifnar1*−/− mice (males 11–12 weeks of age) were infected subcutaneously in the hind feet with GETV (2 × 10^4^ CCID_50_ per mouse). When mice reached or approached ethically defined endpoints (primarily weight loss ≈20%), they were euthanized (†). Control mice were inoculated with heat-inactivated GETV and were euthanized at the same time to provide time matched controls. (**a**) Mice were weighed daily and weight change relative to day 0 is plotted for each mouse. (**b**) Serum viremias for the individual mice described in a. Detection limit ≈2 log_10_CCID_50_/mL of serum (dotted line); ND—not detected. (**c**) Haematocrit (percent) at euthanasia. Three out of five GETV-infected mice had haematocrits greater than those of Controls. Bars represent arithmetic means for each group. See text for statistical treatments.

GETV infection of adult male *Ifnar1*−/− mice resulted in rapid weight loss, which reached or approached the ethically defined endpoint for euthanasia of ≈20% weight loss within 4 days (Figure 1a). The viremias rose very rapidly in GETV-infected mice reaching 8–9 log_10_CCID_50_/mL of serum on days 2–4 post infection (Figure 1b). Similar data were obtained for adult female *Ifnar1*−/− mice (Figure S1).

Increases in haematocrits have previously been reported in mice defective for type I IFN responses infected with CHIKV [41]. Increases in haematocrits are also well established for dengue virus infections in *Ifnar1*−/− mice [71] and in humans [72]. To the best of our knowledge, haematocrit levels have not been reported for lethal GETV infections in piglets. Haematocrit levels in GETV-infected *Ifnar1*−/− mice were thus assessed when they had reached ethically defined endpoints. Overall, GETV-infected mice did not show a significant increase in haematocrit over Controls (Mann–Whitney U, *p* = 0.094), likely in part reflecting the bimodal data distribution in the former group (Figure 1c). Nevertheless, means differed by 7.6 percentage points (95% bootstrap CI: 0.84–13.94 percentage points), with a large effect size (Cliff’s Delta = 0.68; a distribution-free measure appropriate for small samples with unequal variances). In addition, only 12 of 252 possible random arrangements of the data produced a difference of this magnitude (exact permutation test, *p* = 0.048). Taken together, these findings suggest that a biologically meaningful increase in haematocrit can occur during GETV infection in this model.

An elevated haematocrit is a sign of haemo-concentration that can be due to diarrhoea (see below), and can also be a feature of shock or pre-shock states [73], with cytokine storms (see below) able to induce vascular permeability [74,75,76]. The three GETV-infected mice with higher haematocrits also showed elevated levels of endogenous serum lactate dehydrogenase (Figure S2), a non-specific marker of general tissue damage and also an indicator of disease severity in sepsis patients [77].

### 3.2. Findings in GETV-Infected Ifnar1−/− Mice Consistent with Diarrhoea

**Figure 2:**
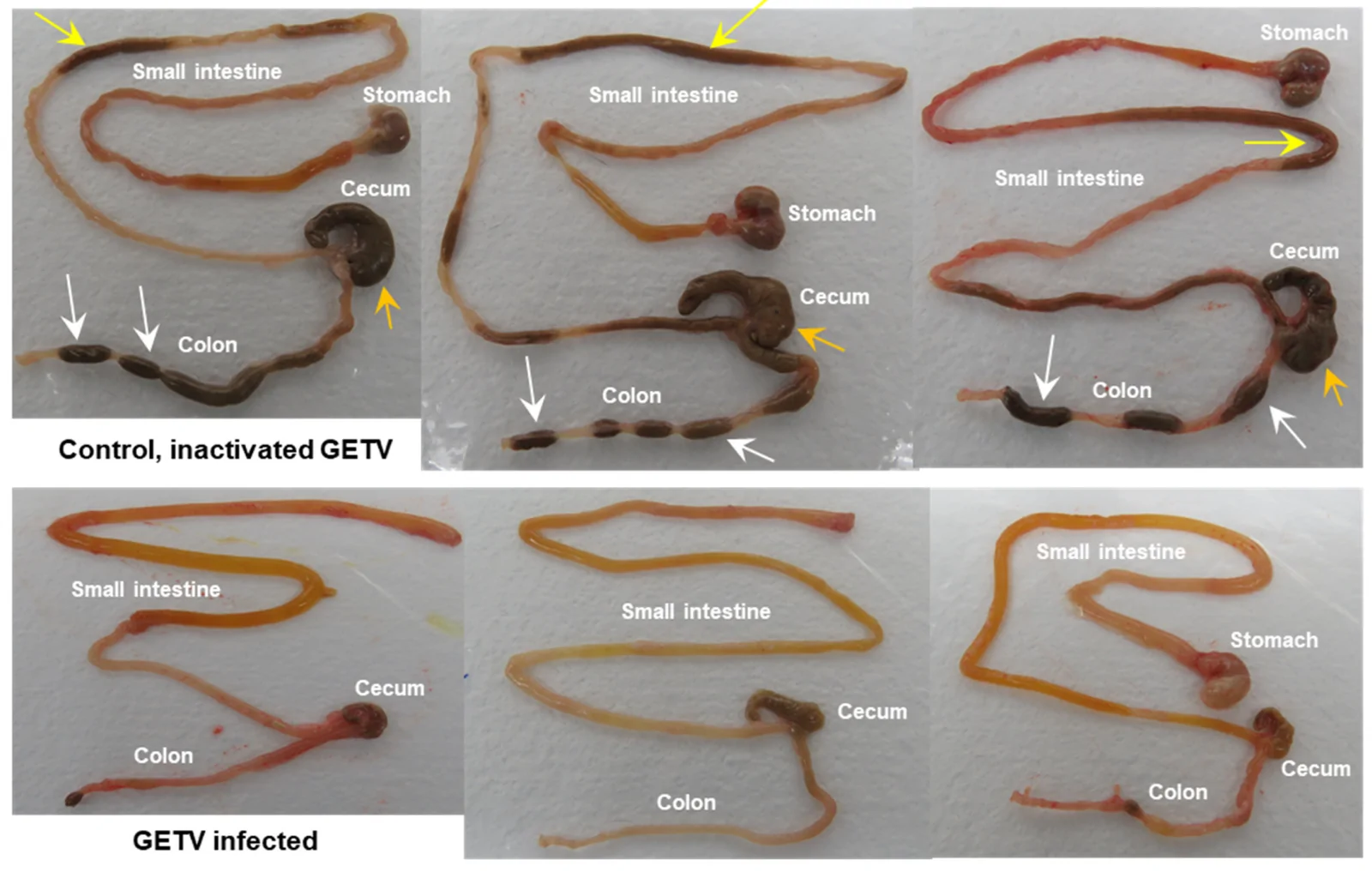
Mouse intestines. The *Ifnar1*−/− mice described in Figure 1 were euthanized on day 3/4 after inoculation with heat-inactivated GETV (Control) or 3/4 days post infection with live GETV. Intestines were dissected out and photographed. Faecal material (dark brown) is clearly visible in the small intestine (yellow arrows), cecum (orange arrows), and colon (white arrows) of Control animals.

GETV-infected mice that had reached the ethically defined endpoints on day 3 or 4 post infection were euthanized (Figure 1a,b), and at the same time, mice that had been inoculated with heat-inactivated GETV (Controls) were also euthanized. As mice were removed from the cage for euthanasia, staff noted loose stools (as distinct from normal hard dry stool pellets) in the wood-shaving bedding of cages housing GETV-infected animals. Intestines were thus dissected out and photographed (Figure 2). Only a limited amount of faecal matter was visible in the intestines of GETV-infected mice. In contrast, Control mice showed normal levels of faecal matter in the small intestine, cecum and colon (Figure 2). These observations are consistent with GETV infection causing diarrhoea in this mouse strain.

Diarrhoea is a non-specific sign/symptom, but is well documented in GETV-infected piglets [6,11,26,27,28,31]. Diarrhoea can also occur during acute infections with other alphaviruses [78], especially in infants and young children [4,79,80].

### 3.3. Splenomegaly in GETV-Infected Ifnar1−/− Mice

**Figure 3:**
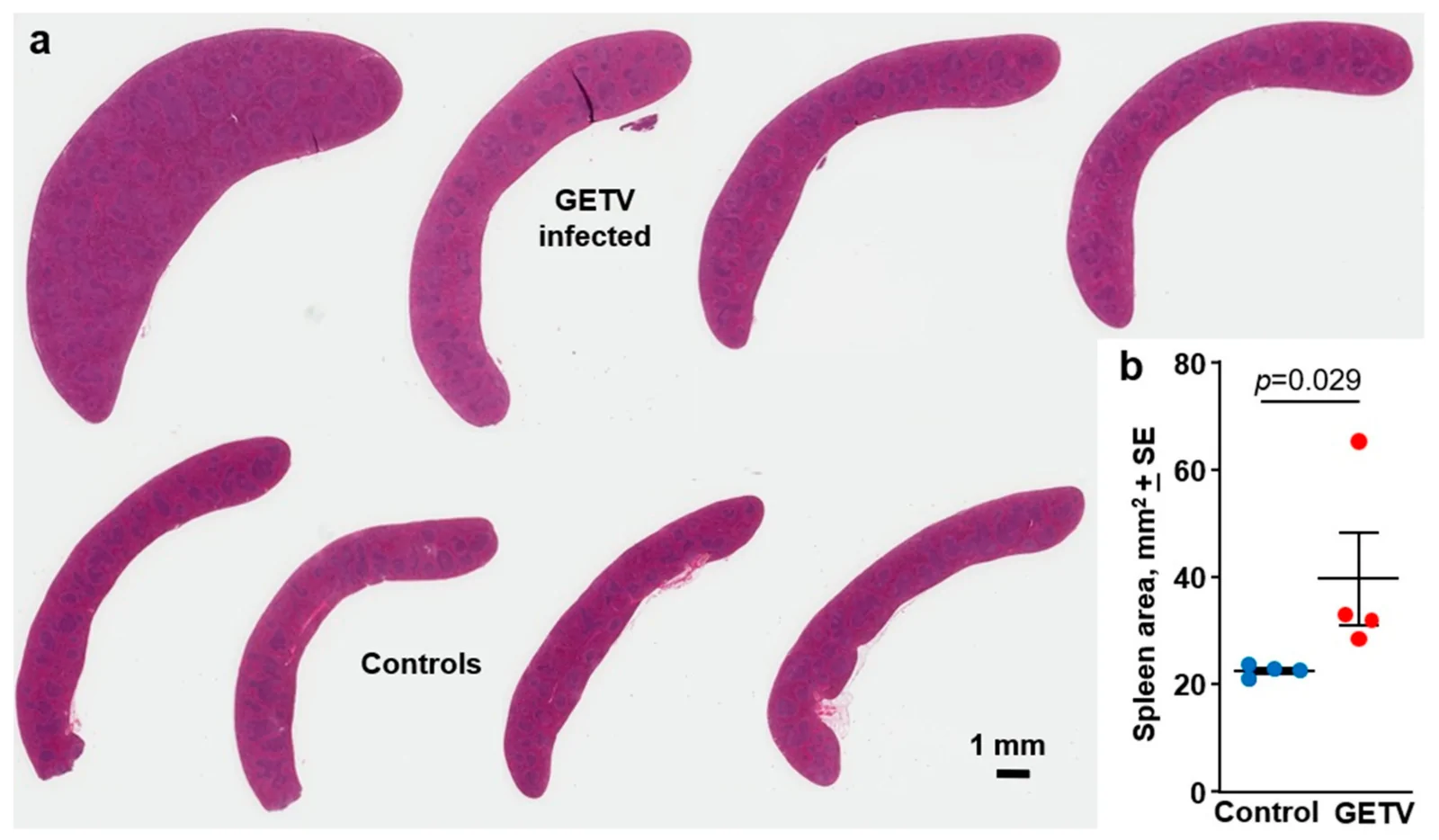
Splenomegaly in GETV-infected *Ifnar1*−/− mice. (**a**) Mice were euthanized on day 3/4 post infection with live GETV (top row, *n* = 4), with Control mice euthanized at the same time points (bottom row, *n* = 4) (mice are described in Figure S1). Spleens were removed, fixed in paraffin and longitudinal median sections were stained using H&E. (**b**) The areas of the stained sections were determined using Aperio ImageScope (annotation window and pen tool). Statistics by Kolmogorov–Smirnov exact test.

Spleens from GETV-infected *Ifnar1*−/− mice (euthanized when they reached ethically defined endpoints on days 3 or 4) and Control mice (euthanized at the same time points) were fixed, embedded in paraffin and longitudinal median sections were stained using H&E. Stained sections were scanned (Figure 3a) and areas determined using Aperio ImageScope (Figure 3b). Overt and significant spleen enlargement (splenomegaly) was seen in GETV-infected animals (Figure 3). Splenic enlargement was reported for piglets experimentally infected with GETV [11]. Reports of splenomegaly associated with infections by other Old World alphaviruses are, to the best of our knowledge, limited to mayaro virus infection of weanling Balb/c mice [81]. Splenomegaly is, however, a feature of dengue virus infections in children [82].

### 3.4. Histopathological Findings in GETV-Infected Mice

**Figure 4:**
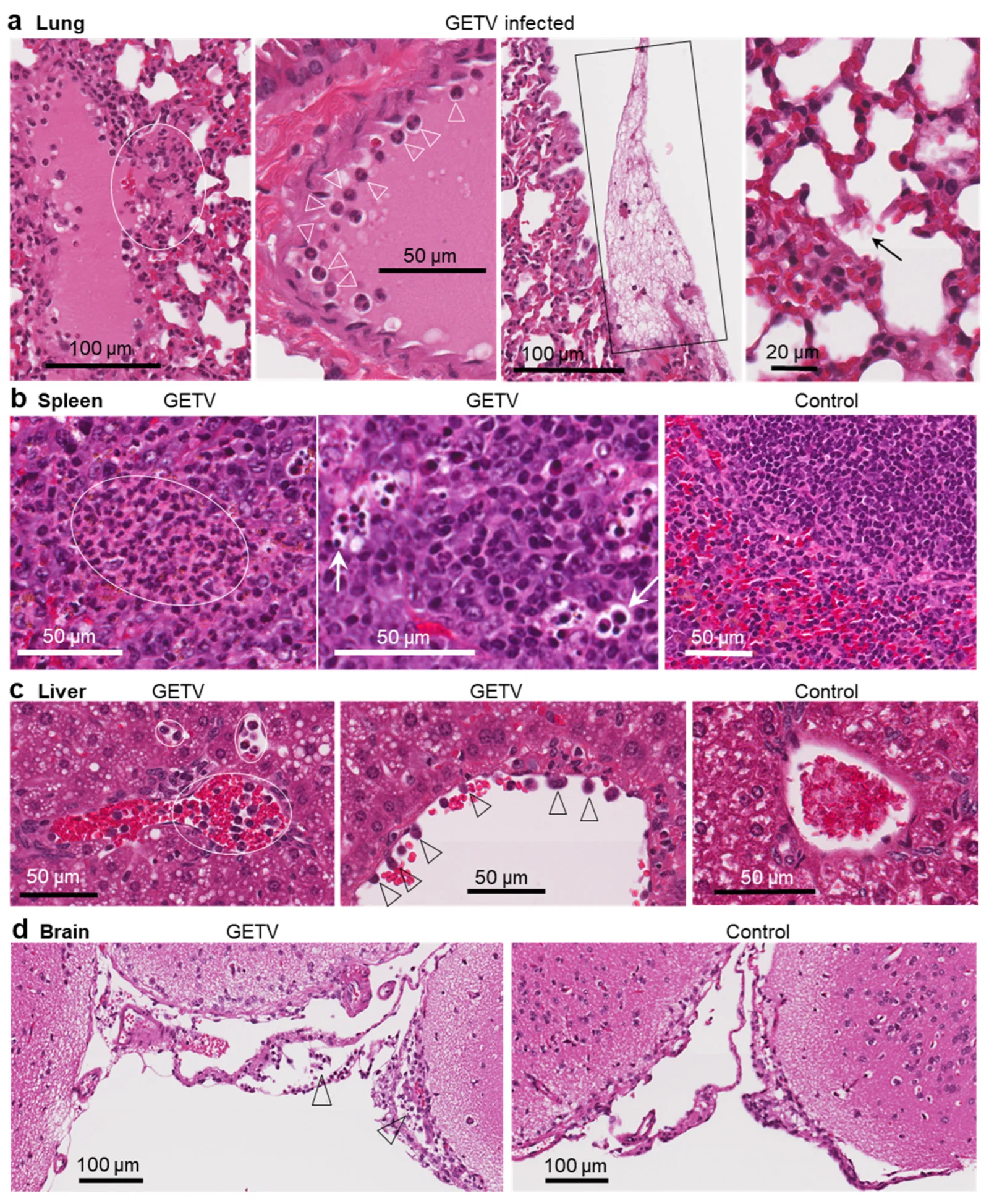
Histopathological features of GETV-infected *Ifnar1*−/− mice. These mice are described in Figure S1 and were euthanized when they reached ethically defined endpoints. (**a**) Lungs showed blood vessel wall damage with leakage (white oval), leukostasis (white arrowheads), fibrinous pleurisy (black rectangle), and fibrin deposits in alveoli (black arrow). (**b**) Spleens showed areas of focal necrosis (white oval) and cells undergoing degeneration (white arrows). (**c**) The liver displayed leukocytosis (white ovals) and leukostasis (black arrowheads). (**d**) The brain showed mild focal inflammatory cell infiltration in meninges (black arrowheads). An expanded description for each mouse and each organ is provided in Table S1.

Tissues from euthanized GETV-infected and Control mice were fixed and embedded in paraffin, and sections were stained with H&E and examined by a European board-certified veterinary pathologist (Figure 4, Table S1).

***Lungs***. The lungs of GETV-infected *Ifnar1*−/− mice showed (i) capillary damage with leakage (Figure 4a, white oval), (ii) leukostasis (Figure 4a, white arrowheads), (iii) fibrinous pleurisy (Figure 4a, black rectangle), and fibrin in alveoli (Figure 4a, black arrow). Although not specifically mentioned, these features do align with histopathology seen in GETV-infected piglets [11,24,27,31]: (i) haemorrhage and leukocyte infiltration in alveolar cavities aligns with damaged capillaries (Figure 4a, white oval) allowing blood into the alveoli; (ii) widening septa and wall thickening align with inflammatory cells (Figure 4a, white arrowheads) causing swelling and wall thickening; and (iii) epithelial desquamation (sloughing of damaged cells) can cause leakage of clotting factors (Figure 4a, black arrow).

***Spleen***. In the T cell areas and marginal zone of the white pulp of the spleen, areas of focal necrosis were observed (Figure 4b, white ovals), with a diffuse increase in the number of cells undergoing degeneration (perinuclear haloing) (Figure 4b, arrow). Similar features were seen in lymph nodes. Descriptions of histopathological lesions in spleens of GETV-infected piglets are limited [27], although one study provided a diagnosis of haemorrhagic splenitis; reduced white-pulp volume, haemorrhage and inflammatory cell infiltration on day 4 post infection [11]. Cellular degeneration (necrosis and apoptosis) generally occurs before white pulp atrophy, haemorrhage and inflammatory cell infiltration, with the latter associated with the clearance of dead cells. Splenic manifestations would appear to be more advanced in piglets than in these mice, although they may reflect the same underlying disease processes.

***Liver.*** The liver of GETV-infected *Ifnar1*−/− mice displayed intravascular leukocytosis (Figure 4c, white ovals) and leukostasis (Figure 4c, white arrowheads), different to blood vessels in Control livers (Figure 4c, Control). Focal infiltration of leukocytes in sinusoidal spaces (blood vessels) and inflammatory cell infiltration in central veins [11] were reported for GETV-infected piglets and align well with the observations in mice. However, the hepatocellular degeneration and haemorrhage reported in piglets [11] was not a finding in mice.

***Brain***. Focal mild inflammatory cells were present in the meninges of GETV-infected *Ifnar1*−/− mice (Figure 4d, white arrowheads). This aligns well with the mild vascular inflammatory cell infiltrate reported for GETV-infected piglets [11]. However, the mild neuron degeneration seen in piglets [11] was not a significant observation in GETV-infected mice.

No significant GETV infection-associated histopathological lesions were identified in the kidneys, intestine or hind feet [41,83]. In summary, histopathological lesions in mice thus appear to be less severe overall than those seen in piglets, with some changes in the liver and brain seen in piglets not observed in *Ifnar1*−/− mice. Nevertheless, histopathology from both species describes severe, acute, multi-organ inflammatory immunopathology.

### 3.5. RNA-Seq and Bioinformatic Analyses of Spleens from GETV-Infected vs. Control Ifnar1−/− Mice

**Figure 5:**
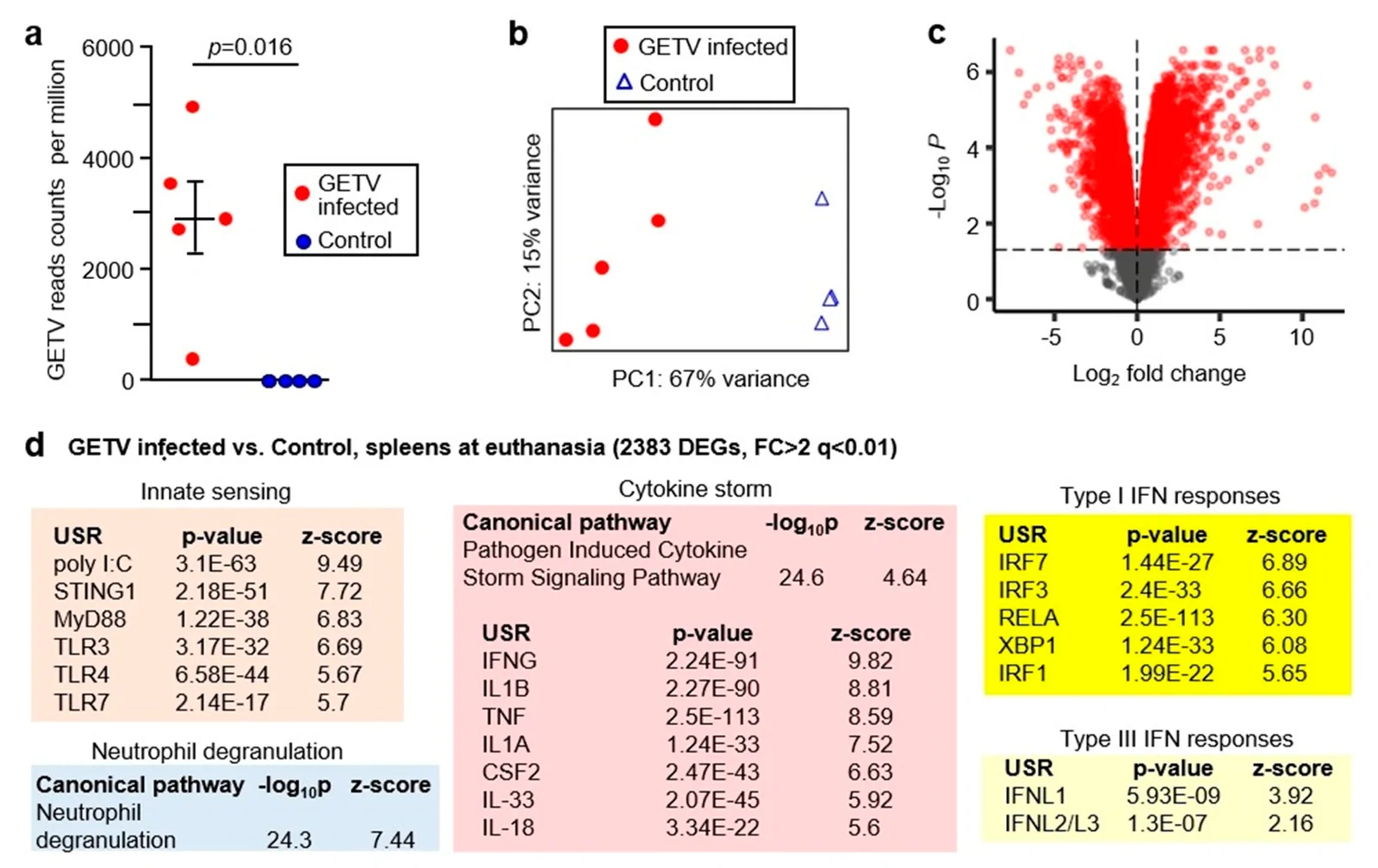
RNA-Seq analysis of whole spleens and a summary of IPA bioinformatic output. (**a**) GETV read counts for spleens from GETV-infected mice and Control mice that were inoculated with heat-inactivated GETV. These are the same mice as shown in Figure 1, with 1 control mouse excluded due to poor RNA quality. Mean and SE are also shown. Statistics by the Kolmogorov–Smirnov exact test. (**b**) Principal Component Analysis plot. (**c**) Visualization of differential gene expression as a volcano plot (GETV infected vs. Control). (**d**) A summary of IPA analyses of 2383 DEGs, with the top scoring annotations boxed into themes. USR—Upstream Regulator. (Full dataset is provided in Table S2.)

At euthanasia, spleens were harvested from GETV-infected mice and at the same time (day 3 or 4) spleens were also harvested from mice inoculated with heat-inactivated GETV (Controls). Spleens were analysed by RNA-Seq and the full dataset is provided in Table S2. Viral read counts illustrated the presence of GETV RNA in the infected mice, with no viral read counts detected in the Controls (Figure 5a). The viral reads in infected mice may reflect virus in blood rather than infection of spleen cells as animals were euthanized at or close to peak viremia (Figure 1b). A Principal Component Analysis (PCA) plot shows a clear separation between infected and control mice (Figure 5b), and a volcano plot illustrates robust and significant changes in gene expression (Figure 5c).

Differentially expressed genes (DEGs) (GETV-infected vs. Control; *n* = 2383, fold change > 2, q < 0.01) were analysed by Ingenuity Pathway Analysis (IPA), with Canonical pathways and Upstream Regulators (USRs) representing primary outputs from such analyses. The latter outputs provide much higher levels of cross-species concordance than comparisons of DEGs [84,85] and are thus the focus of our current report. Canonical and USR pathways are summarized in Figure 5d (full dataset in Table S2). Innate sensing pathways feature prominently, as might be expected for an alphavirus infection [39,42,56,86]. The poly I:C, TLR3 and TLR7 annotations likely reflect detection of viral RNA. The TLR4 annotation may reflect detection of cell debris arising from virus-induced cytopathic effects (CPEs) [87], although gene induction profiles for TLR4 and the other TLRs can overlap [88]. MyD88 is a key signal transduction adaptor for TLR signalling. The STING annotation may reflect detection of mitochondrial DNA from cells undergoing CPEs [89].

Perhaps surprisingly for *Ifnar*−/− mice, robust type I IFN signatures were evident (Figure 5d), with upregulation of many IFNα mRNAs also seen (Figure S3). These type I IFN responses and signatures have also been reported in wild-type mice and humans and are believed to be central for controlling acute alphavirus infections [39,42,56,86,90,91,92]. Alphavirus infections in *Ifnar*−/− mice generally show high viremias and these mice generally succumb to acute infection [42,43]. This is also the case for GETV infection of *Ifnar*−/− mice (Figure 1a) [57], despite these robust type I IFN signatures (see Section 4).

**Table 1:** The association of the top cytokine/chemokine USRs (activation z score > 4, Table S2, cytokine USRs) with references reporting elevated levels of the same cytokine/chemokine in sepsis and/or septic shock. Sepsis/septic shock association with bacterial or viral, or bacterial and viral (Both). (—) No research found specifically associating elevated levels of the indicated cytokine/chemokine with sepsis/septic shock; nevertheless, elevated levels of the cytokine/chemokine may be associated with infections. Elevated levels of cytokines/chemokines determined by measuring protein and/or RNA levels. Literature searches were undertaken with the help of Elicit (Elicit Research, PBC, Oakland, CA, USA), confirmed using Google Scholar, with manuscript downloads via the University of Queensland Library.

UpStream Regulator	z-score	Sepsis association	Sepsis references	Septic shock association	Septic shock references
**IFNG**	9.82	Both	[93,94,95]	Both	[93,96,97,98,99]
**IL1B**	8.81	Both	[94,100,101]. Dengue virus-infected *Ifnar1*−/− mice [102]	Both	[93,103,104,105,106,107]
**TNF**	8.59	Both	[93,94]	Both	[93,108,109,110,111]
**IL1A**	7.52	Both	[112,113]. Signature similar to IL-1B	*-*	Similar to IL-1B
**CSF2** **(GM-CSF)**	6.63	Both	[114,115,116,117,118]	Both	[119,120]
**IL5**	6.47	Both	[121,122]. Severe infections [123]	—	—
**OSM**	6.37	Both	[124,125,126]	Bacterial	[127]
**IL21**	6.29	Viral	[128,129]. Severe COVID-19 [130]	—	—
**IL2**	5.97	Both	[131,132]	Both	[111,133]
**IL33**	5.92	Both	[134,135,136,137,138]	Bacterial	[139]
**IL6**	5.89	Both	[94,140]	Both	[141,142,143,144,145,146]
**IL18**	5.60	Both	[147,148,149,150]	Both	[151,152,153,154]
**PRL** Prolactin	5.53	Both	[155,156,157]	Bacterial	[158,159,160]
**IFNA2/A4**	5.44/4.62	Both	[161] (Not IFNα subtype specific)	Both	[162,163] (Not IFNα subtype specific)
**IL17A**	5.11	Both	[164,165,166,167]	Bacterial	[168,169]. Severe dengue [166]
**IL15**	4.94	Bacterial	[170,171,172]. Virus infection [173,174,175]	Bacterial	[117,176]. Dengue haemorrhagic fever [177]
**EDN1** Endothelin-1	4.91	Both	[178,179]. Severe viral [180]	Bacterial	[181,182,183]
**IFNB1**	4.88	—	Viral and bacterial infections [184,185]	—	—
**TNFSF11 (RANKL)**	4.83	Bacterial	[186]. Virus infection [187]	Bacterial	Higher sRANKL/OPG ratio [188]
**TNFSF12 (TWEAK)**	4.60	Both	[189,190]	Bacterial	[190]
**CCL5 (RANTES)**	4.57	Bacterial	[186,191,192]. Virus infections [193,194]	—	—
**CNTF** Ciliary neurotrophic factor	4.56	—	Viral and bacterial infections [195,196]	—	—
**CSF1 (M-CSF)**	4.20	Bacterial	[197,198,199]. Virus infections [200]	—	—

### 3.6. RNA-Seq of Spleens from GETV-Infected Ifnar1-/- Mice Indicate a Cytokine Storm

Cytokine storms leading to viral sepsis and septic shock are well described for dengue and COVID-19, and are also recognized for a number of other viral infections [201,202,203,204]. IPA analyses provided a top Canonical pathway and top scoring cytokine USRs (Figure 5d) that suggest that *Ifnar1*−/− mice infected with GETV also succumb to a cytokine storm. TNF, IL-1 and IFNγ (Figure 5d, Cytokine storm) are considered archetypal cytokine storm and shock mediators [204,205,206,207], with IFNγ also recognized as a marker of hyper-inflammation [208]. Neutrophil degranulation (Figure 5d) is also a key feature of sepsis [209]. CHIKV infections can cause viral sepsis and septic shock in humans [210] and in *Irf3/7*−/− mice [41], although these are generally considered rare disease manifestations for Old World alphavirus infections [4,5]. A full literature search was thus undertaken, which illustrated that the majority of the cytokine/chemokines identified as top scoring USRs have been reported to be elevated during sepsis and/or septic shock (Table 1). The bacterial sepsis and septic shock cytokine/chemokine literature was included as this is considerably larger and more expansive than the viral sepsis and septic shock literature, with the two (bacterial and viral) showing considerable overlap in upregulated cytokine/chemokine responses (Table 1).

In summary, RNA-Seq of spleens from euthanized GETV-infected and Control *Ifnar1*−/− mice, and ensuing bioinformatic analyses, revealed a pattern of cytokine/chemokine signatures that illustrated a cytokine storm response (Figure 5d), which was strongly indicative of viral sepsis and viral septic shock (Table 1).

## 4. Discussion

The studies reported herein illustrate a series of parallels between GETV-infected *Ifnar1*−/− mice and GETV-infected piglets. As reported previously [57], the high viremias and lethality seen in GETV-infected *Ifnar1*−/− mice (Figure 1a,c) recapitulates the high viremias (up to 7 logs per ml [31]) and lethality seen in GETV-infected piglets. Splenomegaly (Figure 3) has also been reported for GETV infections in piglets [11]. The diarrhoea observed in GETV-infected *Ifnar1*−/− mice (Figure 2) is also seen in GETV-infected piglets [6,11,26,27,28,31], with diarrhoea a common non-specific symptom that can occur during *inter alia* sepsis and septic shock [73]. Overall, the histopathological findings indicate that GETV infection-associated lesions are more severe in piglets than in *Ifnar1*−/− mice, which may reflect the evolutionary adaptation of GETV to preferentially infect ungulates and/or may arise from Australian ethical requirements to euthanize mice before they show unacceptable levels of overt disease. Other factors may also contribute to the lower disease severity observed in *Ifnar1*−/− mice. Detailed histopathology for several organs in GETV-infected piglets relies heavily on one reference [11], and all the reported histopathology (see Section 1) involves contemporary genotype III viruses. Herein, the genotype I MM2021 strain was used, which may be less virulent than genotype III viruses [26,31]. However, direct virulence comparisons in animals are lacking and, as discussed below, virulence phenotypes based on type I IFN antagonism would likely manifest poorly in *Ifnar1*−/− mice. There are also inherent limitations in comparing histopathology in two distantly related mammalian species; mice (superorder *Euarchontoglires*, to which humans also belong) and pigs (superorder *Laurasiatheria*). Nevertheless, histopathological findings for the two species are both indicative of a severe, acute, systemic, multi-organ, inflammatory pathology [211,212,213]. The latter, and especially white-pulp injury and focal lymphoid death in the spleen (Figure 4b), are all consistent with severe sepsis and/or septic shock [214,215].

The RNA-Seq and bioinformatic analyses of cytokine and chemokine responses in GETV-infected *Ifnar1*−/− mice show cytokine storm signatures that further support a diagnosis of sepsis/septic shock (Table 1). To the best of our knowledge, neither protein- nor RNA-based cytokine/chemokine studies have been published for lethal GETV infections in piglets. Only RNA-Seq analyses of in vitro infections of a porcine cell line have been reported [216]. A diagnosis of viral sepsis and/or septic shock has also not (to the best of our knowledge) been formally enunciated or established for GETV-infected piglets in the publicly available literature. Nevertheless, clinical observations associated with lethal GETV infections in piglets [29,31,217] are consistent with viral sepsis and/or septic shock. For instance, cyanosis is well described for piglets with lethal GETV infections [25,27,218] and is a late-stage sign of advanced sepsis or septic shock [73]. Unfortunately, cyanosis is difficult to observe in black mice, which were used here, specifically, *Ifnar1*−/− mice on a C57BL/6J background [62].

Conceivably, GETV infection leads to bacterial or endotoxin translocation from the gut into the circulation (with ensuing endotoxemia-mediated sepsis/shock). To the best of our knowledge, this has never been reported for alphavirus infections, although intestinal damage has been observed by histology for CHIKV infections [219]. No gut histopathology was observed in our study, with diarrhoea often associated with dysregulated ion and water transport in the gut, rather than histologically detectable mucosal injury [220]. Instead, sepsis-associated cytokines can mediate such dysregulated transport in gut epithelial cells [221,222,223], which can then lead to diarrhoea.

The type I IFN receptor is involved in the promotion of bacterial sepsis/septic shock in a number of settings [184,224,225,226]. A similar role for the type I IFN receptor in promoting viral sepsis/shock has not been reported, consistent with the key antiviral (including anti-alphaviral [39]) role played by type I IFN responses. Infections in mice deficient in type I IFN responses often result in high viremias and acute lethality [43], with the data presented herein supporting previous reports [41,227] suggesting that this can lead to viral sepsis/septic shock.

A perhaps surprising observation is the robust type I IFN responses observed in GETV-infected *Ifnar1*−/− mouse spleens (Figure 5d, Figure S3). Why do these responses fail to protect the *Ifnar1*−/− mice from the overwhelming lethal viremia? Cells infected with GETV would be able to generate type I IFN responses via cytoplasmic sensors [44], although these intracellular responses are generally insufficiently fast, robust and/or effective to achieve substantial curtailment of alphaviral replication already initiated in the infected cells [228,229]. Leukocytes can also generate type I IFN responses after TLR-mediated detection of cellular debris and viral RNA released from cells undergoing CPEs [230,231]. The type I IFN receptor mediates a rapid feed-forward loop that rapidly amplifies secretion of IFNαs [232,233], a mechanism clearly absent in *Ifnar1*−/− mice. Nevertheless, consistent with the data presented in Figure S3, IFNαs can still be secreted in vivo in the absence of the type I IFN receptor [234,235,236]. However, the key issue in *Ifnar1*−/− mice is that circulating IFNβ and IFNαs, in the absence of the type I IFN receptor, are unable to protect uninfected cells from rapidly spreading viral infections via induction of antiviral states [237,238]. Thus, even though robust type I IFN responses are seen in whole-spleen tissues, GETV (and other viruses) can spread rapidly in *Ifnar1*−/− mice because IFNα/β, without the type I IFN receptor, is unable to induce antiviral states in uninfected cells [40,239].

Understanding the aforementioned mechanisms may have relevance for the field of oncolytic virotherapy, with GETV being considered a potential treatment for glioblastoma [240]. Viral infection and killing of cancer cells relies on antiviral resistance being induced in healthy cells (via infection-induced circulating IFNα/β), whereas antiviral resistance would need to be less effectively induced in cancer cells [241] to allow for the spread of viral infection through the tumour. Thus, predicting effective GETV virotherapy might focus on tumours that are unable to generate an antiviral state after IFNα exposure. Type I IFN responses induced within GETV-infected glioblastoma cells are perhaps less informative for selecting patients with tumours that might be susceptible to virotherapy [214].

Perhaps perplexing is that genetically identical mice, all inoculated with essentially the same dose of GETV, can show such large mouse-to-mouse variation, for instance, with respect to viral loads (≈2 logs, Figure 1b, Figure 4a and Figure S1), haematocrit changes (Figure 1c) and serum lactate dehydrogenases levels (Figure S2). Age differences between adult mice (beyond 6–8 weeks) may (8 vs. 20 weeks of age [242]) or may not (6 to 12 weeks of age [65]) have a significant influence on alphavirus infection and/or disease in mice. However, infection outcomes for co-caged isogenic mice can actually differ because of social and behaviour factors. Both male and female mice establish social hierarchies [243], wherein subordinate status has been associated with higher basal cortisol [244], which can affect host resistance to viral infection [245,246,247]. In addition, mice held at standard animal house temperatures suffer from a degree of cold stress that can exacerbate viral infections [64,247,248,249]. Huddling can elevate body temperatures [250,251], with the centre of the huddle potentially providing a thermal advantage [251,252]. Social hierarchies are also established in piglets early post partum, with a higher position improving suckling and weight gain [253]. Enhanced early piglet growth has been shown to positively influence innate immunity [254].

Using both male and female mice in our studies (Figure 1, Figure S1) might be viewed as introducing a confounding variable since gender differences in specific cytokine responses following viral infections in humans have been reported, for instance, IL-6 for SARS-CoV-2 infections [255]. At certain challenge doses, some gender differences in viral loads in specific organs can also be seen for CHIKV infection in mice [256]. However, we have been unable to identify studies demonstrating significant male–female differences in alphavirus-induced disease severity or histopathology. In addition, gender differences in GETV infection outcomes for male versus female pigs or piglets have, to the best of our knowledge, not been reported. Also lacking are studies showing different cytokine responses in males versus females during viral sepsis or viral septic shock.

We used the original, widely available MM2021 strain of GETV, rather than more contemporary genotype III viruses that are potentially more virulent [26,31]. Although side-by-side comparisons in wild-type animals are not available, both MM2021 and genotype III viruses showed rapid acute lethality in mice defective for the type I IFN receptor [57,58] (Figure 1a). Virulence in wild-type animals has been linked to the ability of GETV nsP2 to antagonise type I IFN responses [228,257,258], with type I IFN response antagonism generally associated with virulence in alphaviruses [231,259]. Any increased virulence of genotype III viruses, driven by improved abilities to antagonise type I IFN responses, would likely manifest poorly in *Ifnar1*−/− mice, as these mice already have blunted type I IFN responses.

In summary, we have characterized GETV infection in *Ifnar1*−/− mice and proposed that this represents a suitable model of GETV infection in piglets. However, the key contention proposed of viral sepsis/septic shock has yet to be established for lethal GETV infections in piglets, although disease manifestations and histopathology in piglets are consistent with such a diagnosis.

## 5. Conclusions

GETV infections in *Ifnar1*−/− mice recapitulate many of the features seen for GETV infections in piglets. Infections with other Old World alphavirus are primarily associated with self-resolving inflammatory arthropathy [4], which is not recapitulated in *Ifnar1*−/− mouse models [260]. Even if sepsis/septic shock were to be confirmed in lethal GETV infection of piglets, treatment might be viewed as difficult and impractical. Nevertheless, supportive care and intravenous (or intra osseous) fluids may help, given their effective use for treating dengue shock in children [215]. Vaccination of sows with a GETV vaccine [3,22] is perhaps a more feasible option, with protective antibodies [3,23] transferred to the neonates via the colostrum. Vaccination of sows to protect offspring has previously been shown to be effective for an Eastern equine encephalitis virus vaccine [261].

## Data Availability

All relevant data are included within the article and Supplementary Materials. The raw sequencing data generated from the RNA-Seq analysis are publicly accessible from the NCBI Sequence read archive under BioProject PRJNA1517261.

## Supplementary Materials

The following supporting information can be downloaded at https://www.mdpi.com/article/10.3390/pathogens15101079/s1: Figure S1: Infection of female *Ifnar1*−/− mice with GETV; Figure S2 Endogenous serum lactate dehydrogenase; Figure S3. IFNα subtypes and related genes. Table S1: Individual mouse and organ descriptions of histopathological findings associated with GETV infection. Table S2: Full RNA-Seq and bioinformatic dataset.

## Abbreviations

The following abbreviations are used in this manuscript:

GETV	Getah virus
Ifn/IFN	interferon
IRF	interferon response factor
*Ifnar1*	interferon alpha receptor subunit 1 (gene)
CHIKV	chikungunya virus
COVID-19	coronavirus disease of 2019
RNA-Seq	ribonucleic acid sequencing
USR	upstream regulator
mRNA	messenger ribonucleic acid
DEGs	differentially expressed genes
CCID_50_	50% cell culture infectivity dose
TLR	Toll like receptor
CPE	cytopathic effect
H&E	hematoxylin and eosin
