# Supplementary material for: Lethal Viral Sepsis/Septic Shock in Getah Virus-Infected Ifnar1−/− Mice
Source: Pathogens. 2026 Oct 14;15(10):1079. doi: 10.3390/pathogens15101079 (PMC13708548; doi:10.3390/pathogens15101079)
Supplement: Supplementary file 1 [file pathogens-15-01079-s001.zip › Figure S1-S3.pdf]

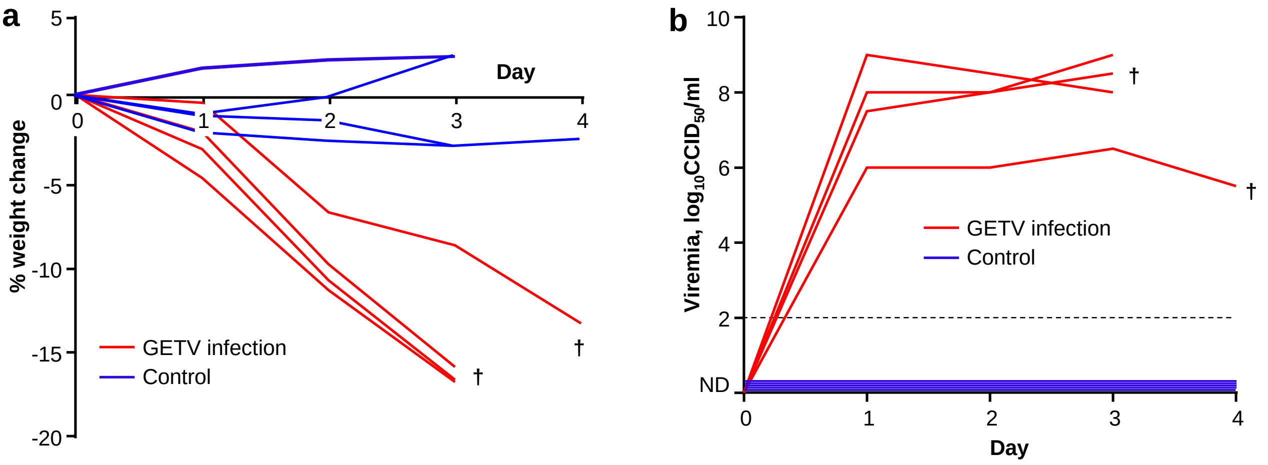


**Figure S1. Infection of female *Ifnar1*-/- mice with GETV**. (**a**) Percentage body weight loss for individual mice infected with 5 $log_{10}CCID_{50}$ GETV (total dose per mouse delivered s.c. in 40 µl tissue culture supernatant per hind foot) (red), or inoculated with the same volume of culture medium, RPMI 1640 supplemented with 2% FCS (Control, blue). n=4 mice per group. In each group the age range was 15-22 weeks, mean age ≈20 weeks. Infected mice were euthanized (†) when ethically defined end points had been reached. (**b**) Individual viremias of the mice shown in a. Limit of detection ≈2 $log_{10}CCID_{50}$/ml (dotted line); ND – not detected.

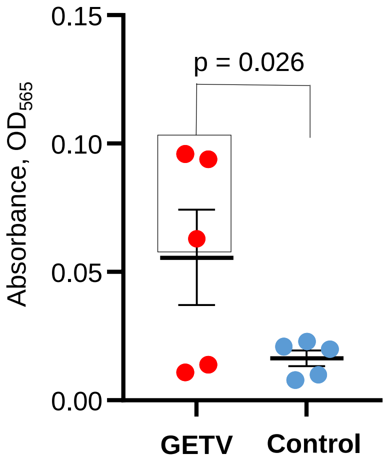


**Figure S2. Endogenous serum lactate dehydrogenase.** Endogenous lactate dehydrogenase (LDH) was measured in serum of euthanized mice using the L-Lactate Assay Kit (Creative Biolabs). All serum samples were heated for 56°C for 1 hr, which does not remove the enzyme activity of LDH1. The GETV-infected mice shown in Figure 1c with a raised hematocrit where the same mice with elevated endogenous serum LDH (boxed). The GETV vs Control groups were not statistically significant for LDH levels. However, the 3 mice with elevated LDH (boxed) showed significantly higher LDH levels compared with Controls; statistics by t test (assuming unequal variance, Welch's t-test).

**a** **Standard filter of 0.25 counts per million**

| GeneID | logFC | PValue | FDR |
|---|---|---|---|
| Ifnar2 | 0.08 | 5.55E-01 | 0.63 |
| Ifnar1 | -0.86 | 4.20E-06 | 6.67E-05 |
| | | | |
| Ifnb1 | 11.39 | 4.52E-05 | 3.48E-04 |
| Ifna2 | 7.79 | 3.30E-08 | 3.50E-06 |

**b** **No filter**

| GeneID | log2FC | PValue | FDR |
|---|---|---|---|
| Ifnar2 | 0.11 | 4.22E-01 | 0.55 |
| Ifnar1* | -0.82 | 4.36E-06 | 9.35E-05 |
| | | | |
| Ifnb1 | 11.40 | 3.31E-06 | 7.56E-05 |
| Ifna4 | 9.04 | 2.99E-05 | 4.05E-04 |
| Ifna5 | 8.64 | 3.76E-05 | 4.79E-04 |
| Ifna1 | 8.40 | 4.95E-05 | 5.93E-04 |
| Ifna | 7.81 | 7.87E-09 | 1.12E-06 |
| Ifna9 | 7.45 | 7.16E-05 | 7.91E-04 |
| Ifna11 | 6.07 | 1.13E-03 | 6.81E-03 |
| Ifna6 | 5.80 | 5.95E-03 | 2.50E-02 |
| Ifna14 | 5.51 | 5.47E-03 | 2.34E-02 |
| Ifna16 | 5.31 | 2.15E-05 | 3.15E-04 |
| Ifna12 | 5.06 | 1.79E-02 | 0.06 |
| Ifna13 | 4.99 | 2.40E-02 | 0.07 |
| Ifnab | 4.70 | 5.96E-05 | 6.85E-04 |
| Ifna15 | 4.28 | 5.08E-02 | 0.13 |
| Ifna7 | 2.04 | 1.64E-01 | 0.31 |
| Ifna-ps1 | 1.36 | 3.66E-01 | 0.50 |

**c**

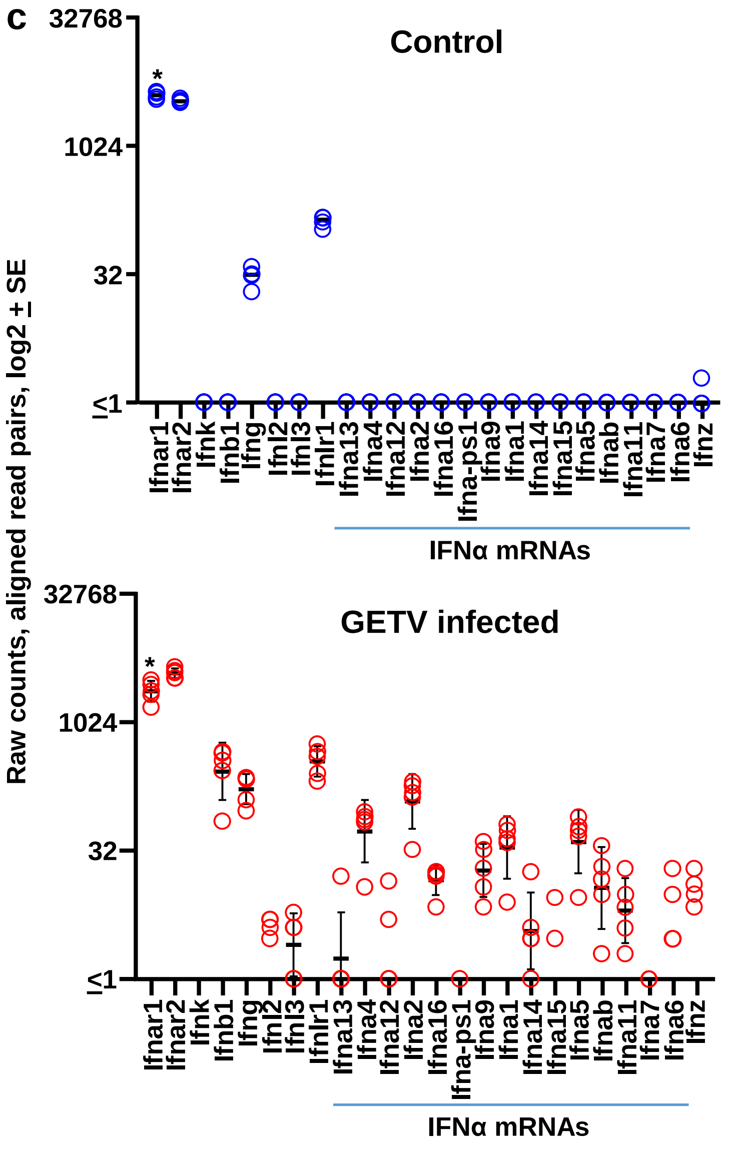


**Figure S3. IFNα subtypes and related genes. (a,b)** When a standard filter is applied to the RNA-Seq data (0.25 counts per million), many of IFNα transcripts are removed as many of these cytokines intrinsically have low mRNA copy numbers (1). When no filter is applied many IFNα genes can be seen to be significantly upregulated (FDR <0.05) in spleen of GETV infected *Ifnar1-/-* mice. FDR >0.05 (not significant) shown by yellow shading. (Full data sets are shown in Table S1). (**c**) The Raw read counts for the same RNA-Seq data set are shown for GETV infected mice (n=5) and Control mice (n=4), again illustrating upregulation of many IFNα genes.

Notes: *Ifnar1 mRNA reads are retained as only one exon is deleted in *Ifnar1-/-* mice, so no functional, full-length protein is made. IFNβ and all IFNαs are thus unable to signal via the type I IFN receptor in *Ifnar1-/-* mice. Although the type I IFN receptor is believed to be central to the feed forward loop that rapidly amplifies secretion of IFNαs, these cytokines can be secreted in the absence of a functional type I IFN receptor (2-4). Ifnar2 transcription is generally only minimally regulated in proinflammatory settings. Ifnar1 and Ifnar2 together form the type I IFN receptor.

**References**


1. Wilson et al *PLoS Pathog* **2017**, *13*, e1006155.
2. Barchet et al *The Journal of Experimental Medicine* **2002**, *195*, 507-516.
3. Kochs et al *Journal of Virology* **2016**, *90*, 9330-9337.
4. Anzaghe et al *Journal of General Virology* **2021**, *102*, 001616.
