# Supplementary material for: Lethal Viral Sepsis/Septic Shock in Getah Virus-Infected Ifnar1−/− Mice
Source: Pathogens. 2026 Oct 14;15(10):1079. doi: 10.3390/pathogens15101079 (PMC13708548; doi:10.3390/pathogens15101079)
Supplement: Supplementary file 1 [file pathogens-15-01079-s001.zip › Table S1.pdf]

Table S1. Individual mouse and organ descriptions of histopathological findings associated with GETV infection.

Main histological features observed in GETV infected mice not seen in Control mice as described by European board certified veterinary pathologist, Dr T Larcher.

GETV infected mouse #1
LUNG alveolar oedema, focal acute pneumonia, leukostasis, fibrin deposits
LIVER perivascular inflammatory cell infiltration
SPLEEN massive lymphocyte degeneration and focal necrosis
LYMPH NODE -
BRAIN focal mild meningeal inflammatory cell infiltration

GETV infected mouse #2
LUNG vascular wall necrosis, alveolar oedema
LIVER -
SPLEEN mild lymphocyte degeneration
LYMPH NODE lymphocyte degeneration and neutrophil infiltration
BRAIN –

GETV infected mouse #3
LUNG vascular wall damage, alveolar oedema, leukostasis
LIVER leukocytosis
SPLEEN massive lymphocyte degeneration and focal necrosis
LYMPH NODE lymphocyte degeneration and neutrophil infiltration
BRAIN non suppurative mild meningitis

GETV infected mouse #4
LUNG pleuritis, fibrinous pleurisy, alveolar oedema
LIVER leukostasis and perivascular inflammatory cell infiltration
SPLEEN marked lymphocyte degeneration and focal necrosis
LYMPH NODE -
BRAIN non suppurative mild meningitis

(-) no significant finding.

Focal infiltration with individual cell necrosis in liver is a non-specific and frequent finding in rodents and a link with virus infection can be excluded. Similarly, tubular dilation with hyalin casts in kidneys is a frequent finding in rodents and a link with virus infection can be excluded.
